# A multi-center study of fidaxomicin use for *Clostridium difficile* infection

**DOI:** 10.1186/s40064-016-2825-x

**Published:** 2016-08-02

**Authors:** Dhara N. Shah, Fay S. Chan, Nandita Kachru, Krutina P. Garcia, Holly E. Balcer, April P. Dyer, John E. Emanuel, Michelle D. Jordan, Katherine T. Lusardi, Geri Naymick, Radhika S. Polisetty, Lanny Sieman, Ashley M. Tyler, Michael L. Johnson, Kevin W. Garey

**Affiliations:** 1 Pharmacy Practice and Translational Research, University of Houston College of Pharmacy, 1441 Moursund Street, Houston, TX 77030 USA; 2 Pharmaceutical Health Outcomes and Policy, University of Houston College of Pharmacy, Houston, TX USA; 3 Department of Pharmacy, Roper St Francis Healthcare System, Charleston, SC USA; 4 Department of Pharmacy, Southeastern Health, Lumberton, NC USA; 5 Department of Pharmacy, OhioHealth MedCentral Hospitals, Mansfield, OH USA; 6 Department of Pharmacy, Vidant Medical Center, Greenville, NC USA; 7 Department of Pharmacy, UAMS Medical Center, Little Rock, AR USA; 8 Department of Pharmacy, St. Mary’s Hospital, Madison, WI USA; 9 Department of Pharmacy, Northwestern Medicine Central DuPage Hospital, Winfield, IL USA; 10 Department of Pharmacy, North Kansas City Hospital, North Kansas, MO USA; 11 Department of Pharmacy, Saint Thomas Midtown Hospital, Nashville, TN USA

**Keywords:** Pharmacoepidemiology, Anaerobe infections, Real-world study, Treatment, Clinical practice

## Abstract

**Purpose:**

Fidaxomicin use in real-world clinical practice, especially for severe *Clostridium difficile* infection (CDI), is mainly based on single-center observational studies. The purpose of this pharmacoepidemiology study was to assess outcomes of patients given fidaxomicin based on episode number and use of concomitant antibiotics.

**Methods:**

Fidaxomicin use over time across included hospitals in the United States was assessed using a large inpatient drug utilization database. A multicenter retrospective chart review was also conducted of hospitalized patients with CDI that received fidaxomicin between 2011 and 2013. Fidaxomicin utilization and clinical outcomes were stratified by use of fidaxomicin for first or second episode (early episodes) versus greater than or equal to episodes (later episodes).

**Results:**

The overall fidaxomicin use rate was 2.16 % which increased from 0.22 % in the last two quarters of 2011 to 3.16 % in the first two quarters of 2013. A total of 102 hospitalized patients that received fidaxomicin from 11 hospitals were identified in the multicenter study. Sixty-nine patients received fidaxomicin for early (68 % with severe CDI) and 33 received for later episodes. The majority of patients received other CDI therapy including 61 patients (88 %) for early episodes and 27 (82 %) for later episodes. Concomitant non-CDI antibiotics were received by 48 patients (47 %). Rates of clinical outcomes were similar regardless of CDI episode.

**Conclusion:**

This study demonstrated a slow but steady increase in fidaxomicin utilization over time; most of which was combined with other systemic antibiotics. Antimicrobial stewardship teams should provide guidance on appropriate use of fidaxomicin to optimize therapy and assess the need to continue other antibiotics during CDI treatment.

## Background

According to the Centers for Disease Control and Prevention (CDC), the annual incidence of CDI in the United States exceeds 500,000 hospitalized cases with 29,000 deaths (Lessa et al. 2015). Treatment options for CDI are limited. Metronidazole and oral vancomycin have been utilized as the mainstay therapy for CDI (Kelly and LaMont 2008; Cohen et al. 2010). However, morbidity and mortality including the recurrence of CDI continues to be a major concern with recurrence rate up to 25 % after discontinuation of treatment (McFarland et al. 1999). Moreover, patients with at least one recurrence have a higher probability of experiencing further recurrences (Sheitoyan-Pesant et al. 2016). CDI is also associated with increased healthcare costs due to longer hospitalizations and re-hospitalizations (Lawrence et al. 2007; Kyne et al. 2002).

Fidaxomicin, a narrow spectrum macrocyclic antibiotic, displays many favorable qualities including inhibition of spore formation (Babakhani et al. 2012), preservation of intestinal microbiota (Louie et al. 2012), and reduced acquisition of VRE and candida in patients treated with fidaxomicin (Nerandzic et al. 2012). In two, large phase III studies, fidaxomicin was shown to decrease recurrent CDI compared to oral vancomycin in patients given the drug during their first occurrence or first recurrence of CDI disease (Louie et al. 2011; Cornely et al. 2012a, b; Crook et al. 2012). The current practice guidelines by the Society for Healthcare Epidemiology of America (SHEA) and Infectious Disease Society of America (IDSA) 2010 clinical practice guidelines (Cohen et al. 2010) was published prior to the Food and Drug Administration (FDA) approval of fidaxomicin. The European Society of Clinical Microbiology and Infectious Diseases (ESCMID) guidelines have mentioned fidaxomicin as one of the mainstay CDI therapy and is recommended as one of the options for initial, non-severe CDI, initial, severe CDI, first recurrent CDI, and in patients at a risk for recurrent CDI based on limited evidence (Debast et al. 2014).

A case study published after its approval reported limited benefit of fidaxomicin when used for multiple recurrent CDI (Orenstein 2012). Two other studies have demonstrated positive benefits on the use of fidaxomicin using a pre-defined protocol (Goldenberg et al. 2016; Gallagher et al. 2015). Despite these findings, there seems to be a lack of consensus on the optimal time to utilize fidaxomicin for CDI management. Furthermore, data regarding fidaxomicin utilization in real-world clinical practice. The objective of this study was to evaluate fidaxomicin use nationwide in the real-world clinical setting. The specific aims of the study was (1) to evaluate fidaxomicin use and overall utilization rate stratified by regions, hospital location, and hospital type and CDI therapy utilization in the United States; (2) to evaluate fidaxomicin use based on CDI episode number, use of other CDI therapy and concomitant non-CDI antibiotic use.

## Methods

The use of fidaxomicin was assessed using a nationally representative database and a multisite, chart review study of all patients receiving fidaxomicin. For specific aim 1, the Premier Perspective Database (Safavi et al. 2014) was used to identify patients with CDI. Premier Perspective Database is a largest inpatient drug utilization database, consists of data from a network of approximately 3400 United States hospitals, including >45 million inpatients and >200 million outpatients. Data from 372 hospitals contain date-stamp log of all billing files that includes drug billing data at the individual patient level. The database encompasses events during hospitalizations such as diagnoses and medication administration information directly from electronic health records. Each encounter of a patient is given a unique identifier. Data collection included information from the third quarter of 2011 to the second quarter of 2013. Data from CDI patients based on ICD-9 code (008.45); type of *Clostridium difficile* testing; number of patients that received fidaxomicin; location of hospital (rural vs. urban); hospital type (teaching vs. non-teaching); length of hospital stay (in days); duration of fidaxomicin therapy; use of other CDI antibiotics for the same episode (metronidazole, vancomycin, rifaximin); and basic demographics such as age, gender, and race were collected.

For specific aim 2, a multicenter review of each hospital’s electronic health record was conducted. Participating hospitals collected data on adult patients (age >18 years) with CDI that received fidaxomicin between June 2011 and June 2013. Eligible participants were identified through the practice-based research network of making a difference in Infectious Diseases (MAD-ID) (http://mad-id.org/the-mad-id-research-network/). CDI was defined as positive fecal *C. difficile* toxin EIA or PCR test plus diarrhea and/or other signs and symptoms of CDI. The first documented occurrence of CDI was classified as CDI episode 1 and subsequent episodes were labeled as 2, 3, 4, 5, etc. The severity of CDI as mild-moderate versus severe was determined for patients with first or second episode of CDI. A case of CDI was considered severe if admitted to an intensive care unit (ICU), presence of pseudomembranous colitis based on colonoscopy or presence of any two of the following parameters such as age >60 years, temperature >38.3 °C, albumin <2.5 mg/dL, or WBC >15,000 cells/mm^3^ (Zar et al. 2007). Details regarding fidaxomicin regimen, standard regimen (200 mg PO twice a day) or a different regimen along with start and stop dates of fidaxomicin were recorded. The use of other CDI therapy for the same CDI episode in which fidaxomicin was utilized was categorized as subsequent, subsequent and combination, or combination therapy. Subsequent CDI therapy was switching to another CDI therapy for the management of same CDI episode. A change to a combination therapy in which one agent utilized was fidaxomicin after starting on ≥1 CDI therapy was labeled as subsequent and combination. Combination therapy included initiation of fidaxomicin plus another CDI therapy at the same time. The use of non-CDI antibiotics received during the CDI therapy with fidaxomicin, concomitant non-CDI antibiotics, was documented as a dichotomous variable. The utilization of concomitant proton-pump inhibitors, histamine-2 (H2) receptor antagonist, and immunosuppressant agents such as any systemic steroids, tacrolimus, mycophenolate, sirolimus, cyclosporine, chemotherapy agent was also documented. For patients that received multiple episodes of fidaxomicin, data from the first documented use of fidaxomicin for the patient at the institution was primarily collected. Episode of fidaxomicin use was stratified by first or second episode (early episodes) and compared to greater than or equal to three episodes (later episodes).

Clinical cure was defined as cure documented in the health electronic record or discharge to home or discharge to a site that requires resolution of diarrhea prior to admission. Other clinical outcomes including recurrent CDI, re-hospitalization and all-cause mortality within 90-days after the initiation of fidaxomicin use were evaluated. Another CDI episode after initial resolution of CDI episode based on positive fecal *C. difficile toxin* EIA or PCR test and/or signs/symptoms of CDI was defined as recurrent CDI. Re-hospitalization was any re-admission to the index hospital. CDI recurrence during the re-hospitalization was considered CDI-related re-hospitalization and all other re-hospitalization was due to other co-morbidities as a primary reason for re-hospitalization.

### Ethics, consent, and permissions

The study was approved by institutional review board at the University of Houston (CPHS:2365) and each participating site. A waiver of consent was granted due to the retrospective, de-identified nature of the data collection. All data were reported in aggregate and individual patient data were not reported.

### Data analyses

Using the Premier database, the overall fidaxomicin use (%) was determined by dividing the number of patients that received fidaxomicin by the total number of CDI patients during the given time period. The fidaxomicin use (%) data was stratified by geographic region; West, Mid-West, Northeast and South. Fidaxomicin use was also compared by hospital variables (hospital location in rural versus urban area and hospital type, teaching versus non-teaching hospitals). Descriptive statistics used to evaluate characteristics of the cohort that received fidaxomicin. The average duration of fidaxomicin therapy and average length of hospital stay (in days) per the number of patients that received fidaxomicin were evaluated among this cohort. Chi squared test was used for inferential statistics of categorical data. Two-tailed tests were used and a *p* value <0.05 was considered significant. Using data from the multicenter, chart review study, treatment patterns of fidaxomicin in relation to other CDI antibiotics were assessed. Demographics, formulary status, and study objectives were tabulated and summarized. Usage of fidaxomicin was stratified by episodes and usage patterns and outcomes were reported globally as well as by episode number.

## Results

A total of 474 patients received fidaxomicin among 21,924 patients with CDI diagnosis based on the ICD-9 code 008.45 for intestinal infection with CDI. The overall use rate of fidaxomicin among all patients with CDI was 2.16 % (Table 1). The fidaxomicin utilization rates increased from 0.22 % in the last two quarters of 2011 to 3.6 % in the first two quarters of 2013 (p < 0.0001). Stratified by geographic regions, fidaxomicin utilization rate ranged from 1.91 to 2.51 % with small variations noted among the regions (West region having the highest rate and the Northeast region with the lowest rate). Out of 2577 patients with CDI in the rural area hospitals, 38 patients (1.47 %) received fidaxomicin compared to 436 of 19,347 patients (2.25 %) in the urban area hospitals (p = 0.01). Fidaxomicin use rates were similar for teaching (2 %) and non-teaching (2.27 %) (p = 0.18).

**Table 1:** Fidaxomicin utilization rates

	2011 Quarters 3 and 4	2012 Quarters 1 and 2	2012 Quarters 3 and 4	2013 Quarters 1 and 2	Overall time period
Fidaxomicin utilization from third quarter of 2011 to second quarter of 2013					
Number of CDI patients	5098	5582	5160	6084	21,924
Number of patients who received FDX	11	119	126	218	474
FDX use rate	0.22 %	2.1 %	2.4 %	3.6 %	2.16 %

	West	Mid-West	South	Northeast
Fidaxomicin utilization rates stratified by regions in the United States				
Number of CDI patients per region	3143	5050	9026	4705
Number of patients who received FDX per region	79	105	200	90
Overall FDX use rates per region*	2.51 %	2.08 %	2.21 %	1.91 %

	Hospital location		Hospital type	
	Rural	Urban	Teaching hospitals	Non-teaching hospitals
Fidaxomicin utilization rates stratified by hospital location or hospital type				
Number of CDI patients per hospital location or type	2577	19,347	9304	12,620
Number of patients who received FDX per hospital location or type	38	436	187	287
Overall FDX use rates per hospital location or type*	1.47 %	2.25 %**	2 %	2.27 %^

*CDI* *Clostridium difficile* infection, *FDX* fidaxomicin

* Combined data for the entire time period; ** p value = 0.01 rural hospitals versus urban hospitals; ^ p value = 0.18 teaching versus non-teaching hospitals

Out of the 474 patients that received fidaxomicin, two were excluded from this analysis as they were under the age of 18 years. The average age of the 472 patients that received fidaxomicin was 70 ± 15 years and 65 % were females. Three-hundred seventy-five (79.4 %) were Caucasians, 10.6 % were African-American and 10 % were other. The majority of patients had CDI listed as the primary (44 %) or secondary (51 %) diagnosis. The majority of patients were tested for *C. difficile* using enzyme immunoassay (EIA) for *C. difficile* toxin(s) (50 %) or polymerase chain reaction (PCR) (49 %). For the management of CDI, 26 patients (5.5 %) received fidaxomicin as monotherapy. The remaining 446 patients received metronidazole (n = 55), oral vancomycin (n = 101) or a combination of metronidazole plus vancomycin (290) for the same episode. It is unclear if the other CDI therapy were received as subsequent or combination therapy. Rifaximin was also received by 22 patients along with fidaxomicin and other CDI therapy. The average duration of inpatient fidaxomicin therapy was 6 ± 4 days. The average total length of hospital stay was 13 ± 11 days.

A total of 102 patients (average age: 68 ± 16; females: 59 %; Caucasian: 68 %) with CDI that received fidaxomicin were evaluated in the multicenter chart review study from 11 US hospitals. Hospital types were either academic-based teaching (n = 7) or community-based non-teaching (n = 4). Common co-morbidities in this cohort include hypotension (57 %), renal disease (29 %) and diabetes (28 %). Twenty-five patients (25 %) had other gastrointestinal disorders (such as chronic diarrhea or constipation, diverticulitis, irritable bowel syndrome, or ulcer disease). Twenty-one patients (21 %) had malignant solid tumor and six patients (6 %) had leukemia/lymphoma. All patients had received previous CDI therapy included metronidazole, vancomycin PO, or a combination of metronidazole (intravenous or oral) and oral vancomycin for patients with greater than or equal to two episodes either during a previous episode or at an earlier time period in the treatment course.

Fidaxomicin was a non-formulary medication at 6 (n = 54 %) of hospitals. A fidaxomicin use protocol or order set with specific criteria was implemented in two hospitals. In the absence of a formal protocol, two other hospitals reported involvement of an antimicrobial stewardship pharmacist in confirming that fidaxomicin was utilized for recurrent CDI or in patients that have clinical failure after metronidazole or oral vancomycin use. At most hospitals, including where fidaxomicin was non-formulary (n = 8), fidaxomicin was restricted to a specialty consult service (Infectious diseases (ID) consult (7); ID or gastrointestinal (GI) consult (1)).

Fidaxomicin was used at a similar proportion for a patient’s first (n = 37), second (n = 32) or third or more (n = 33) episode of CDI (Table 2). Out of 69 patients treated during an early CDI episode, 22 patients (32 %) had mild-to-moderate CDI and 47 patients (68 %) had severe CDI. Fidaxomicin at a standard dose of 200 mg by mouth twice daily was utilized for all patients with an inpatient therapy duration range of 1–28 days (average: 9 ± 4 days). Ninety-nine of 102 patients were initiated on fidaxomicin as an inpatient, of which 22 patients were in the ICU. The three patients that received outpatient fidaxomicin therapy received other CDI therapy while in the hospital. Total of 48 patients (47 %) received concomitant non-CDI antibiotics. Concomitant PPIs were utilized by 47 % (n = 48), H2-receptor antagonists by 15 % (n = 15), and immunosuppressant agents by 18 % (n = 18). Thirteen patients (13 %) received fidaxomicin monotherapy for the episode of CDI and 88 patients received other CDI therapy for the same episode in which they received fidaxomicin. Out of 88 patients, 48 patients received subsequent therapy, 16 received subsequent and combination therapy, and 4 received combination therapy. For the remaining 20 patients, type of therapy was not able to be determined from the medical chart. Among patients that received subsequent or subsequent and combination CDI therapy, 15 patients were initiated on fidaxomicin and 49 patients were initiated on metronidazole (n = 8), oral vancomycin (n = 14) or oral vancomycin plus metronidazole (n = 27). Subsequently, these patients were either switched to another CDI agent or a combination therapy, including fecal transplantation in two patients. Total of four patients received combination therapy in which other CDI therapy was started at the same time as fidaxomicin.

**Table 2:** Fidaxomicin utilization stratified by CDI episodes

	Early episodes			Later episodes	Overall (n = 102)
	Episode 1 (n = 37)	Episode 2 (n = 32)	Total (n = 69)	Episode ≥ 3 (n = 33)	
Mild-moderate CDI; n(%)	10 (27 %)	12 (37.5 %)	22 (32 %)	Not applicable	22/69 (32 %)
Severe CDI; n(%)	27 (73 %)	20 (62.5 %)	47 (68 %)	Not applicable	47/69 (68 %)
1. FDX monotherapy; n (%)	3 (8 %)	4 (12.5 %)^a^	7 (10 %)	6 (18 %)	13 (13 %)
2. Other CDI therapy; n (%)	34 (92 %)	27 (84 %)	61 (88 %)	27 (82 %)	88 (86 %)
I. Subsequent; n	18	14	32	16	48
II. Subsequent and combination; n	8	6	14	2	16
III. Combination; n	2	1	3	1	4
IV. Unable to categorize; n	6	6	12	8	20
Concomitant non-CDI antibiotics; n (%)	25 (68 %)	10 (31 %)	35 (51 %)	13 (39 %)	48 (47 %)

CDI = *Clostridium difficile* infection; FDX = fidaxomicin

^a^For one patient: unknown if received fidaxomicin as monotherapy or with other CDI therapy

The length of hospital stay ranged from 1 to 90 days (mean ± SD: 19 ± 19 days). Clinical outcomes stratified by CDI episode number are found in Table 3. The overall clinical cure rate was 77.4 % (n = 79). Specifically, the cure rate was 68 % for patients with CDI episode 1, 77 % for patients with CDI episode 2, and 88 % with CDI episode 3 or greater. Forty-four of 49 patients (90 %) that completed therapy with fidaxomicin only had clinical cure compared to 19 of the 33 patients (57.6 %) that completed therapy with another CDI therapy or a combination that included fidaxomicin with another CDI antibiotic. The recurrence rate within 90-days after initiation of fidaxomicin and after the initial resolution of CDI episode was 21 % (n = 21), which included six patients with episode 1, seven patients with episode 2, and eight patients with episode 3 or greater. Thirty-two of 102 patients were re-hospitalized within 90-days of receiving fidaxomicin (episode 1: 28 %; episode 2: 41 %; episode ≥3: 31 %). Among the patients that were re-hospitalized, 14 of 32 patients had documented CDI recurrence during the re-hospitalization. Eight of 102 patients died within 90 days of receiving fidaxomicin (Episode 1: four patients; episode 2: four patients). Three patients died within 7 days, two died within 21 days, and three died between 30 and 90 days after fidaxomicin initiation.

**Table 3:** Clinical outcomes stratified by CDI episodes

n	Early episodes		Later episodes	Overall (n = 102) n (%)
	Episode 1 (n = 37)	Episode 2 (n = 32)	Episode ≥ 3 (n = 33)	
Clinical cure	25	25	29	79 (77 %)
Recurrent CDI	6	7	8	21 (21 %)
Re-hospitalization^a^	9	13	10	32 (31 %)
Mortality	4	4	0	8 (8 %)

^a^Out of 32 re-hospitalizations, 14 patients had documented CDI recurrence during that re-hospitalization

For patients that required treatment for a further episode of CDI, a number of treatment strategies were used. Fidaxomicin use was given for further CDI episodes for 16 patients of whom 13 received one further treatment course and 3 received two further courses. For these further treatment courses, fidaxomicin was given as monotherapy (n = 5), as subsequent therapy or combination therapy with metronidazole or oral vancomycin (n = 7), or fidaxomicin followed by vancomycin taper (n = 2), or fecal microbiota transplantation (n = 2). Further episodes that did not contain fidaxomicin were generally oral vancomycin-based regimens.

## Discussion

In 2011, fidaxomicin was approved for the treatment of CDI. Prior to this time, metronidazole and oral vancomycin had been the mainstays of therapy. Despite demonstrating decreased CDI recurrence rates compared to vancomycin, fidaxomicin use was generally thought to be low likely due to a high drug acquisition price and perhaps due to an under-recognition of the importance of recurrent CDI (Aitken et al. 2014). This study is the first, nationally representative trial to assess global fidaxomicin use throughout the USA. Using the Premier Perspective Database, it was demonstrated that fidaxomicin use has increased slowly but steadily since its introduction with no specific area of the country or hospital type adopting increased fidaxomicin use more than others. Using a multicenter, medical chart review study, it was demonstrated that fidaxomicin is used somewhat equivalently based on CDI episodes but is almost universally associated with either other CDI antibiotics or other non-CDI systemic antibiotics. This scattered use may be explained by non-formulary status of fidaxomicin, hospitals reserving this drug for a certain patient populations, or lack of guidance from the clinical practice guidelines (Cohen et al. 2010; Bauer et al. 2009). However, optimal use of this narrow-spectrum antibiotic is likely diminished by use of other antimicrobials that kill microbiota (Mullane et al. 2011). These results provide immediate opportunity to antimicrobial stewardship efforts to optimize use of fidaxomicin. Strengths of the study include a combination of a large, nationally representative database along with a multicenter, chart review study that included academic and community-based hospitals.

The majority of patients in this study (86 %) received other CDI therapy for the management of the same CDI episode in which fidaxomicin was used. A high rate of receipt of other CDI therapy has been previously shown in other real-world fidaxomicin studies. Despite having a specific guideline for use, Eiland et al. reported that 13 of 60 (22 %) patients received other CDI therapy during the same episode specifically sequential therapy of metronidazole followed by fidaxomicin (13.3 %) or a combination therapy of metronidazole plus fidaxomicin (8.3 %) (Eiland et al. 2015). Vargo et al. studied 61 patients that received fidaxomicin (severe CDI: 31 %; recurrent CDI: 53 %), of whom the majority (>50 %) received fidaxomicin therapy in a combination with at least one other CDI therapy usually as sequential therapy after receiving another CDI therapy (Vargo et al. 2014). In a small case series of 15 transplant patients that received fidaxomicin (recurrent CDI: 40 %), 7 patients (47 %) received fidaxomicin as salvage therapy after receiving metronidazole, oral vancomycin, or both (Clutter et al. 2013). These results agree well with our findings in which the majority of patients also received other CDI antibiotics during the same treatment episode. Although the reasons for this high rate of use of other CDI antibiotics during the same treatment episode is unclear, it can be posited that this may be due to a difficult to treat patient population in which treatment options are limited and perhaps due to an underappreciation of the importance of a healthy microbiota for the sustained treatment of CDI.

Clinical response and recurrence rates in this study also agree with smaller case series. Unlike previous studies, in this current study, we stratified patients by severity based on the method suggested by Zar et al. based on previous studies that baseline or premorbid creatinine may not be available for many patients; a requisite for severity stratification using the SHEA/IDSA guidelines (Cohen et al. 2010; Zar et al. 2007; Shah et al. 2013). In an evaluation of 50 patients that received fidaxomicin for salvage therapy (severe CDI: 26 %; severe, complicated: 22 %; recurrent CDI: 54 %; concomitant, non-CDI antibiotics: 72 %) (Penziner et al. 2015) the clinical cure rate was 64 % (Penziner et al. 2015). In a smaller observational study of 15 transplant patients, the clinical cure rate was 67 % (Clutter et al. 2013). Two other similar case series demonstrated clinical cure rate that ranged from 72 to 97 % (Eiland et al. 2015; Vargo et al. 2014). These cure rates are lower than what was observed the phase III clinical trials and likely reflects a sicker patient population than what was enrolled in the indication trials (Louie et al. 2011; Cornely et al. 2012). These results also agree well with result from Mullane et al. who reported a lower clinical cure rate (84 %) and a higher recurrence rate (25 %) in a post hoc analysis of the phase III clinical trial in which 27.5 % patients received concomitant non-CDI antibiotics (Mullane et al. 2011). The recurrence rate within 90 days after initiation of fidaxomicin treatment in our study was 21 % which agrees well with the study by Mullane et al. Taken together, these results provide strong evidence that the anti-recurrence properties of fidaxomicin are likely negated if another broad spectrum antibiotic either directed towards *C. difficile* or other bacterial pathogens are included in the same treatment course. Investigations on novel uses of fidaxomicin such as taper or chase therapy are being conducted and may lead to more optimal use of this drug. However, we would suggest that concomitant use of fidaxomicin with broad-spectrum CDI antibiotics during the same treatment course be limited and discouraged (Soriano et al. 2014). This advice follows from two recently reported studies that demonstrated decreased CDI recurrence and subsequent re-hospitalizations following a formulary change to fidaxomicin (Goldenberg et al. 2016; Gallagher et al. 2015). In both of these studies, fidaxomicin was instituted earlier in the disease course as suggested monotherapy with benefits noted. It is likely that similar stewardship efforts will be required in these populations to achieve the same benefit.

This study has several limitations. This study collected data up to 2013 thus analyses will have to be repeated as fidaxomicin becomes more widely adopted. Although, we used a nationally representative database and a multicenter study design, we will not have identified all usage patterns for fidaxomicin. The retrospective chart review provided granular details regarding CDI therapy but not all data was available or may have been recorded. Likewise, the reasons for choice of therapy and appropriateness of concomitant antibiotics were not able to be assessed. Diagnostic test for CDI vary between sites making inter-site comparisons difficult. Recurrence rates were based on medical chart review thus patients with CDI recurrence that did not have this information in the medical chart would have been missed. However, this data along with previous investigations provide strong evidence that optimization of fidaxomicin in patients with CDI is required in order to get the full benefit from the drug. Antimicrobial stewardship programs should specifically target appropriate use of fidaxomicin in their list of priorities.

## Conclusion

 This study demonstrated a slow but steady increase in fidaxomicin utilization over time. The majority of patients receive fidaxomicin with either other CDI-directed antibiotics or other systemic antibiotics. This likely decreases the effectiveness of fidaxomicin, a narrow-spectrum antibiotic. Antimicrobial stewardship teams should provide guidance on appropriate use of fidaxomicin to optimize therapy and assess the need to continue other antibiotics during CDI treatment.
